# Volume Twelve

**Published:** 1872-08

**Authors:** 


### Editorial.

### Volume Twelve.

The beginning of a new volume, to the Editor of a Medical Journal, is something like the beginning of a new year, or even of a new life to people in general; it is, at least, suggestive of thought and reflection. The first desire which it naturally stimulates is that the new be better than the old, and various plans are laid to promote this end. The profession have favored us exceedingly, and we have been able to publish some of the most instructive and valuable papers which have appeared in this country, and alas! we have also published some which were of no value whatever, "worth a little less than nothing." It was anciently deemed best to let the wheat and tares grow together, and we have acted somewhat upon the principle of the Divine teaching, but we mean to have as few tares, and as much wheat as possible. Our readers will appreciate the impossibility in our climate, of having a perfectly clear crop, "free from weeds." Our Journal has been open to varied expression of opinion, and our attentive readers must have noticed that our aim was to offer a free medium of communication with the profession.

Another train of reflection is also stimulated by the yearly steps, as they are taken. The early volumes of our Journal were commenced, as some will remember, at a time of such political and financial depression and distress, that according to all human calculation, its completion could not be expected, but the clouds and darkness have passed away and our subscribers have learned that if the Journal is late in its issue, it is the fault of the printer, and not due to the death of the Journal.

For the future, the whole field of Medical knowledge open for cultivation, and the entire medical profession invited to enter in and labor, we offer such as is gathered, believing that with the continuance of former favors we shall send out a volume of which the profession may be proud.

